# Pain and Sleep Biomarkers in Participants Undergoing Orthopedic Surgeries

**DOI:** 10.3390/ijms26135959

**Published:** 2025-06-21

**Authors:** Manish Bhomia, Nicholas A. Giordano, Krista B. Highland, Keren Lee, Matthew Van Shufflin, Yanru Feng, Alexandra Kane, Raymond B. Kroma, Barbara Knollmann-Ritschel

**Affiliations:** 1 Department of Pathology, Uniformed Services University, 4301 Jones Bridge Road, Bethesda, MD 20814, USA; fengyanru@gmail.com (Y.F.); barbara.knollmann-ritschel@usuhs.edu (B.K.-R.); 2 Henry M. Jackson Foundation for the Advancement of Military Medicine, Bethesda, MD 20817, USA; alexandra.kane.ctr@usuhs.edu; 3 Nell Hodgson Woodruff School of Nursing, Emory University, 1520 Clifton Road, Atlanta, GA 30322, USA; nicholas.a.giordano@emory.edu; 4 Department of Anesthesiology, Uniformed Services University, 4301 Jones Bridge Road, Bethesda, MD 20814, USA; krista.highland@usuhs.edu; 5 Department of Anesthesiology, Naval Medical Center Portsmouth, 620 John Paul Jones Cir, Portsmouth, VA 23708, USA; keren.lee323@gmail.com; 6 Department of Anesthesiology, Brooke Army Medical Center, 3551 Roger Brooke Drive, Fort Sam Houston, TX 78234, USA; mattvanshufflin@gmail.com; 7 Drexel University College of Medicine, 60 North 36th Street, Philadelphia, PA 19104, USA; bobbykromamed@gmail.com

**Keywords:** pain, sleep, biomarkers, cytokines

## Abstract

The bidirectional relationship between chronic pain and poor sleep are well reported. Disrupted sleep and chronic pain, either alone or in conjunction, are often associated with poor post-surgical outcomes. However, the relationship between peripheral blood biomarkers and chronic pain and sleep disturbances after orthopedic surgery has not been extensively studied. The goal of this observational prospective study was to conduct an analysis on the relationship of blood cytokines and chemokines with chronic pain and sleep outcomes among US service members undergoing orthopedic surgery. Active-duty service members (N = 114) who underwent orthopedic extremity or spinal surgery were recruited, of whom 69 completed pre-surgery and 64 completed 6-week post-surgery surveys and blood draws. Blood cytokine and chemokine analyses were performed using multiplex immunoassays. Non-parametric correlations with blood cytokine and chemokine showed significant associations with both pre- and post-surgical pain scores whereas no significant correlations were observed with sleep disturbance scores. Increased pain intensity 6 weeks after surgery was positively associated with increased hepatocyte growth factor (ρ_s_ = 0.11; *p* < 0.05) and negatively correlated with interleukin-2r (ρ*_s_*= −0.42; *p* < 0.001). This study found that inflammatory biomarkers are associated with pre- and post-surgical pain but not sleep disturbances.

## 1. Introduction

Sleep disturbances [1] and pain [2] are independently associated with reduced functioning and quality of life. Mitigating the compounding effects of disrupted sleep and pain on subsequent health outcomes are of particular interest in optimizing the care of patients undergoing orthopedic surgeries. Between 40% and 89% of patients requiring orthopedic surgery experience poor sleep quality [3]. Up to a third of patients undergoing arthroscopic procedures develop chronic pain [4]. The synergistic, and at times bidirectional, relationship between pain and sleep in individuals with acute and chronic pain have been well-documented [5]. Sleep disturbances and pain may result in a cascade of inflammatory responses, lending support for common underlying biomarkers to be leveraged in predicting the development of poor post-surgical outcomes. However, research is needed to examine how underlying blood and plasma-based markers correlate with patient-reported symptomatic outcomes related to sleep and pain.

There is evidence to suggest that there are potential biomarkers of sleep disturbances and pain in the post-surgical period [6], such as TNF-alpha. However, less information is known regarding the degree to which these biomarkers are uniquely associated with each symptom, or whether similar biomarkers underlie both sleep disturbance and pain. In a non-surgical sample with cancer, pro-inflammatory biomarkers (e.g., IL-2, IL-6, IL-12, TNF-alpha, interferon gamma, GM-CSF) were associated with reductions in sleep quality after undergoing oncological therapies, but chemokines and anti-inflammatory biomarkers were not [6]. Whereas, in a sample of older patients undergoing laparoscopic hepatobiliary surgery, peripheral inflammatory markers were associated with perioperative sleep disturbances, but varied between pre-surgery (neutrophil-to-lymphocyte ratio, systemic Immune-inflammation index, IL-6, IL-10) and post-surgery (neutrophil-to-lymphocyte ratio, systemic immune-inflammation index, IL-10) sleep disturbances [7]. In regards to post-surgical pain, one study of biomarkers collected within 24 h after surgery found 3-month chronic pain intensity was associated with CC-X-C motif chemokine ligand 10 (CXCL10) [8] and another found neutrophil-to-lymphocyte ratio changes were associated with a higher incidence of chronic post-surgical pain a year after surgery [9]. Thus, while it is possible that pain intensity and sleep disturbances could have similar pro-inflammatory biomarkers and chemokines, there is a lack of data exploring their potential common links in a surgical sample.

Much of the research examining the bidirectional nature of pain and sleep, and the underlying inflammatory pathway, has been conducted in lab-based settings [10] or with animal models [11]. Few studies have examined potential biomarkers shared by poor sleep and pain presentations in clinical populations undergoing common orthopedic procedures. Even fewer studies have sought to elucidate the link between variations in inflammatory response markers and subsequent changes in longitudinal patient-reported outcomes following surgery. Despite the need to identify biomarkers linked to pain and sleep to guide future therapies, substantial barriers to biomarker-based research are significant, including cost, time, and challenges with supply chains [12]. As such, preliminary research can support future targeted research by providing the strength of relationships between biomarkers of interest and symptoms, as well as the extent to which they may change before and after surgery.

The preponderance of research has focused on examining biomarkers uniquely linked to either sleep or pain [12]. To date, much of the research jointly examining the association between sleep and pain presentations after surgery has been limited to cross-sectional investigations or has been conducted with patients seen in oncologic settings [13,14]. Limited longitudinal research incorporating biomarkers and patient-reported outcomes has been conducted with service members undergoing orthopedic surgeries despite being a patient population that experiences a higher rate of injury, sleep problems, and pain compared with civilian populations [15]. The post-surgical period presents an opportune time to study underlying inflammatory markers linked to patient-reported outcomes, as this is a particularly critical point, both for potential transition to chronic pain and potential prolonged opioid therapy [16]. Studies linking objective biomarkers to patient-reported outcomes in the post-surgical period are needed to guide future resource-intensive research that may benefit from ascertaining both clinical and biological endpoints to study therapeutic benefits in surgical patient populations. Based on the existing literature, our working hypothesis for this study was that individuals undergoing orthopedic surgery will exhibit significant changes in specific plasma biomarker levels (such as inflammatory cytokines and chemokines) from pre-surgical to post-surgical periods, and these changes will be correlated with the magnitude of change in self-reported sleep disturbance and pain scores. This observational prospective study sought to test this hypothesis in service members prior to and after orthopedic surgery.

## 2. Results

### 2.1. Sample Characteristics

Of the 114 participants enrolled in this study, 71 had at least a single time point (baseline and/or 6-week follow-up) in which they completed both patient-reported outcomes and provided blood samples; 62 had both patient-reported outcomes and biomarker data across both time points. At baseline, 111 participants completed the patient-reported outcomes, 69 of whom also provided blood samples. At the 6-week follow-up, 75 participants completed patient-reported outcomes, 64 of whom also provided blood samples. Participants who completed patient-reported outcomes but not blood samples at baseline (n = 45) did not significantly vary from participants who provided both blood samples and patient-reported outcomes at baseline (n = 69) across age, race and ethnicity, assigned sex, American Society of Anesthesiologists (ASA) level, body mass index, military service length, Patient Reported Outcome Measurement Information System (PROMIS) sleep disturbance, or pain intensity; but those with both blood and patient-reported outcomes at baseline had a disproportionate proportion of participants who underwent spinal surgery (26% versus 5%; *p* = 0.02).

For participants included in the analysis, the median age was 38 years [IRQ 32, 48]. Most participants identified as non-Latine white (68%), followed by Black (13%), or another race and ethnicity (7%); 8 participants (12%) had missing race and ethnicity data. Most participants had an assigned sex of male (77%) and an ASA Level I or II (94%). The median body mass index was 28.4 [IQR 25.3, 31.2] and median length of military service was 16 years [IQR 6, 23]. Most participants received procedures on an upper extremity (39%; 1 elbow ulnar nerve transposition, 1 shoulder hydrodilatation, 2 shoulder arthroplasty with unknown open or arthroscopic approaches, 10 arthroscopic, and 13 open procedures), followed by a knee (26%; 6 open and 12 arthroscopic knee procedures), the spine (26%; 3 single-level fusions, 3 two-level fusions, and 12 microdiscectomy or laminectomy procedures), or a hip (9%; all 6 arthroscopic procedures).

### 2.2. Change in Patient-Reported Outcomes and Biomarkers

From pre- to post-surgery, there were noted changes in patient-reported outcomes and biomarkers, as indicated by paired sample median difference tests with false discovery rate adjusted *p*-values (Table 1, Supplementary Table S1). For example, pain intensity scores decreased between time points, whereas PROMIS sleep disturbance scores remained stable. In follow-up analyses, the majority of participants did not experience a minimal clinically important difference (e.g., 2 points) in pain intensity scores (71%), whereas 20% reported a decrease of more than 2 points and the remaining 9% reported an increase. Relatedly, most (75%) participants had less than a 5-point change in PROMIS sleep disturbance scores, while 14% had at least a 5-point decrease and 11% had at least a 5-point increase. From pre- to post-surgery, chemokine ligand 9 (CXCL9) and vascular endothelial growth factor A (VEGF-A) significantly decreased, whereas IL-1 significantly increased.

### 2.3. Association Between Patient-Reported Outcomes and Biomarkers

Both pre- and post-surgery, several biomarkers significantly correlated with pain intensity but not sleep disturbance scores, per false discovery rate adjusted *p*-values (Table 2, Supplementary Table S2). Before surgery, pain intensity was positively associated with epidermal growth factor (ρ = 0.24), IL-1-beta (ρ = 0.38), IL-4 (ρ = 0.30), recombinant human IP-10 (ρ = 0.34), CXCL9 (ρ = 0.29), CCL3 (ρ = 0.28), and CCL4 (ρ = 0.31). At 6-weeks post-surgery, pain intensity was negatively correlated with IL-2 receptor (ρ = −0.421).

## 3. Discussion

In this exploratory observational study of US active-duty service members undergoing orthopedic surgery, biomarkers, specifically inflammatory markers, were associated with pain intensity before and 6 weeks after surgery, however sleep disturbance scores did not correlate with the biomarker expression before and after surgery. In particular, two interleukins (IL1-beta, IL-4) and four chemokines (CXCL10, CXCL9, CCL3, CCL4) were associated with increased pain intensity before surgery but, after surgery, increased pain intensity scores were associated with lower interleukin-2 receptor levels.

This study builds upon previous cross-sectional research indicating interleukins are associated with pain outcomes. The present findings are similar to prior research that has demonstrated the relationship between IL-1-beta, pain, and sleep [13,14], albeit it was only associated with pre-surgical pain in the present study. Moreover, in the present study, IL-4 was associated with pre-surgical pain. Similarly, in a sample of participants living with knee osteoarthritis, pain relief was mediated by changes in IL-1ß and IL-13 [17]. IL-4 has also been associated with disc degeneration. Intervertebral disc cells isolated from patients when treated with IL-4 resulted in suppression of the release of pro-inflammatory factors in response to lipopolysaccharide (LPS) stimulation [18,19]. Additionally, both IL-4 and IL-13 have been consistently associated with arthritis and implicated in the downregulation of the inflammatory process, thereby associated with reduced pain in one literature review [20].

In this study, IL-17A was significantly and positively associated with pre-surgical pain intensity without adjustment, but the false discovery rate adjusted *p*-value was not significant. A review of IL-17A literature demonstrated a reliable relationship with various forms of pain, including inflammatory, neuropathic, and chronic pain [21], as well as disc degeneration [22]. Additionally, IL-17A levels have been reportedly higher in patients living with osteoarthritis, relative to those without a pain condition [23]. One interleukin, IL-2 receptor, was negatively correlated with pain intensity after surgery. Prior research demonstrated that participants who underwent surgery for traumatic musculoskeletal injury had increased levels of IL-6, as well as IL-8 and IL-16, but decreased levels of IL-12 [24].

Overall, the present results build upon previous studies and suggest a relationship between serum cytokines and chemokines with pain in participants who have undergone orthopedic surgery. IL-1ß is a cytokine that induces downstream chemokines such as CXCL10 and CCL3. In the present study, both CXCL10 and CCL3 were associated with pain. CXCL10, a chemokine produced by neurons in response to inflammatory stimuli [25], and CCL3 have been shown in the literature to be strongly associated with several types of chronic pain including neuropathic pain in both animal models and in humans [26,27]. Moreover, CCL3 [28] has been associated with intervertebral disk degeneration. CXCL9 may be elevated in patients who have chronic pain conditions, such as fibromyalgia [29]. While IL-6 has been associated with post-surgical pain [30] and IL-6, IL-8, and TNF-alpha have been associated with lumbar radicular pain [31], the finding did not replicate in the present study, but, instead, other interleukins were associated with pain intensity.

The complex and dynamic nature of pain presentations necessitates the continued discovery and validation of cytokines linked to pain and sleep disruptions after surgery. It is unclear the extent to which the biomarkers in this study are prognostic and could be used to evaluate therapeutic response assessment (e.g., indicator of early response to pain therapy or a mediator of clinical endpoints). However, linking objective biomarkers to patient-reported outcomes after orthopedic surgery, as was carried out in this hypothesis generating study, is a crucial component to the continued effort for the clinical validation of reliable generalizable markers [12]. The results could be further verified in animal models, where cytokine expression in the brain can be studied to determine whether there is a central implication. The lack of association between biomarkers and sleep disturbance scores may be due in part to the change in sleep presentations throughout the post-surgical period. Sleep can be improved through a range of evidence-based non-pharmacological [32] and pharmacological modalities [33]. As such, continued research on biomarkers specific to sleep disturbances, to include both subjective and objective sleep assessment, after orthopedic surgery is warranted.

This study had several inherent limitations. The small sample size, variability of the biospecimens and potential timing of sample collection, heterogenous surgical procedure representation, and suboptimal follow-up rates were limiting. For example, this preliminary study was not designed to capture potential diurnal variation in biomarker expression [34] or account for sample collection timing, which may be important for future studies. The limited sample size likely resulted in inadequate power to detect statistically significant effects with *p*-value adjustment for multiple comparisons. A more homogenous surgical sample may have resulted in lower variability and increased ability to detect statistically significant effects. The lack of follow-up may have led to selection bias in those that remained in this study. Additionally, not all patient-reported outcomes (e.g., acute distress) [35] and clinical factors (e.g., medications) were captured in this observational study and could be associated with changes in the biomarkers and outcomes that may be important to consider in future studies. Lastly, this study did not include a subsample of participants who were not undergoing orthopedic surgeries. Future studies could incorporate a variety of patient populations to include patients with acute and chronic pain conditions, those without, and those undergoing surgeries.

Given that the present study of active-duty service members undergoing orthopedic surgeries was more homogenous than the overall US active-duty population and civilian patient populations, generalizability is limited. For example, 2022 estimates indicated that the majority of service members (65%) are 30 years of age and younger, whereas the median age in the present sample was 38 years [36]. Further, the observational nature of this work limits our findings to being associative in nature, not causal. Therefore, future research is warranted to determine how cytokines may drive pain outcomes, or pain experiences, including related stress response, may drive inflammatory marker levels. Given the preliminary scope of this study, it is unclear whether the present findings are clinically meaningful. Despite these limitations, this study’s sample and follow-up period extend the understanding of how plasma-based peripheral biomarkers are linked to patient-reported outcomes throughout post-surgical recovery. Specifically, this study’s sample size and follow-up period extend on earlier investigations on patients undergoing orthopedic surgery [37,38].

This prospective observational study of service members undergoing orthopedic surgery showed that inflammatory linked biomarkers are associated with varying pain presentations but not sleep disturbances. Specifically, IL-1-beta, IL-4, CXCL10, CXCL9, CCL3, and CCL4 levels significantly correlated with pre-surgical pain, while post-surgically IL-2 receptor levels were negatively associated with pain intensity up to six weeks after surgery. These results illustrate the utility of pairing changes in validated patient-reported outcome measures to objective biomarkers up to six weeks after surgery as potential objective markers of recovery and improvements in symptoms. Exploring pathways linked to the biomarkers found in this study could be useful for future research focused on developing novel pain therapies that address underlying biological processes. Continued validation studies of these markers and their association with longitudinal post-surgical patient-reported outcomes are warranted.

## 4. Materials and Methods

### 4.1. Participants and Procedures

**Figure 1:**
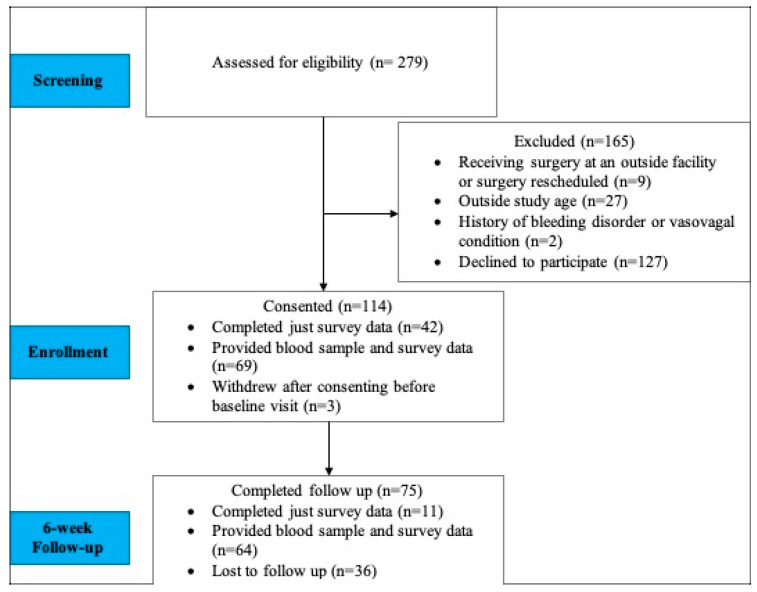
Patient flow diagram representing the clinical recruitment, including the inclusion and exclusion criteria.

This observational prospective study was approved by the Walter Reed National Military Medical Center Institutional Review Board (WRNMMC-2018-0132). All study procedures were in accordance with the institutional guidelines and approved IRB protocol. Informed consent was obtained from all the participants prior to enrolment in this study. Participants were eligible to participate in this study if they were an adult (ages 18–65) active-duty service member undergoing orthopedic procedures of the upper extremity (e.g., labrum repair, rotator cuff repair, biceps tenotomy/tenodesis, and/or distal clavicle resection), lower extremity (e.g., anterior cruciate ligament reconstruction, meniscus repair, and cartilage procedure), or spine (e.g., microdiscectomy, spinal fusion) at Walter Reed National Military Medical Center who could complete the informed consent requirement and study procedures. This study also required HIPAA authorization to review medical record information. Once enrolled, participants completed pre-surgical and 6-week post-surgical surveys and blood draws. This follow-up time point was selected to reflect the clinical practice workflow, as this was when participants commonly returned to the hospital for post-surgical visits (Figure 1).

### 4.2. Variables of Interest

Patient-level factors: Covariates collected from surveys included years of age, self-reported assigned sex, and service duration; Patient Reported Outcome Measurement Information System (PROMIS) computer adaptive testing (CAT) item banks included sleep disturbance [39] and physical function [40]. Each PROMIS item bank was administered with 4–5 questions, on average, and provided T-scores (range = 0 to 100, mean = 50, standard deviation = 10). Higher scores on the PROMIS sleep disturbance measure indicated higher symptom burden and sleep problems whereas lower scores on the PROMIS physical function measure indicated lower function. Previous studies of patients undergoing spine surgery found that a 3.5 [41] to 7.4 [42] point change in PROMIS sleep disturbances may be a minimally important difference, therefore a threshold of a 5-point change was selected. Current pain intensity was assessed with the 0–10 Defense and Veterans Pain Rating Scale, with higher scores indicating greater pain [43]. A threshold of a 2-point change in pain intensity indicated a potentially clinically meaningful threshold [44]. Race and ethnicity, ASA physical status classification, body mass index, and surgery type (upper extremity, lower extremity, spine) were collected by study staff from electronic health records.

Biomarkers: A panel of 30 human cytokines and chemokines were analyzed using Human 30 plex procartaplex array (Thermo Fisher Inc, Waltham, MA, USA) as per the manufacturer’s recommendations. A 100 µL serum was used for this analysis and an ELISA assay was conducted using the recommended kit. The assay plate was read on a Bioplex 100 plate reader (BioRad Inc, Hercules, CA, USA). The exported data was analyzed for using the ThermoFisher cloud connect utility application. These cytokines and chemokines were chosen given their known association with sleep and pain in other clinical populations [45,46,47].

### 4.3. Statistical Analyses

All analyses were completed using R Statistics (https://www.r-project.org/ accessed on 1 September 2024). First, paired median difference tests evaluated the extent to which patient-reported outcomes and biomarkers changed before and after surgery; a standardized mean difference between time points was calculated using the rstatix R package [48]. Next, non-parametric correlations (ρs) evaluated the strength of the relationships between patient-reported outcomes and biomarkers within each timepoint. The false discovery rate method was used to calculate adjusted *p*-values for multiple outcomes using the stats and bcdstats R packages [49]. Across all analyses, no imputation was performed to account for missing data

### 4.4. Power Analysis

Power analyses were calculated for paired median and correlation tests using the pwr R package [50]. Due to the lack of complete data, the presumed paired median sample size was set at 62, with a significance level of 0.05, 80% power, and the assumption was a two-sided test. Estimates indicated that there was an adequate sample size to detect an effect size (Cohen’s d) of at least 0.36 between paired samples. For correlations, the presumed sample size was 69 at baseline and 64 at the 6-week follow-up, with 0.05 significance level and 80% power. Estimates indicated that there was adequate power to detect and effect size (correlation coefficient) of at least 0.33 and 0.34 at baseline and 6-week follow-up, respectively.

## 5. Conclusions

The findings in this manuscript demonstrated significant associations between blood-based biomarkers and pre- and post-surgical pain intensity but not sleep disturbances. These biomarkers can be used as indicators for tracking clinical recovery and assessing pain-related symptoms.

## Figures and Tables

**Table 1:** Change in patient-reported outcomes and biomarkers prior to and after surgery.

Patient-Reported Outcome or Biomarker	Pre-Surgery Median [IQR]	Week 6 Follow-Up Median [IQR]	SMD of Change
Pain Intensity	4 [3, 6] **	3 [2, 5]	0.42
PROMIS Sleep Disturbance	54.3 [47.63, 59.6]	54.3 [50.4, 59.4]	0.08
PROMIS Physical Function	41.8 [36.8, 47.0] *	38.8 [34.4, 44.7]	0.29
Eotaxin (CCL-11)	101.2 [52.6, 165.9] *	82.8 [67.6, 108.2]	0.24
Fibroblast Growth Factor 2 (FGF-2)	0 [0, 7.0] *	0 [0, 4.2]	0.26
Granulocyte Colony-Stimulating Factor (CSF3)	6.8 [0, 27.2]	24.8 [0, 39.4] *	0.30
IL-1 Receptor A	240 [170, 318] *	208 [158, 296]	0.25
IL-6	5.6 [1.5, 16.0] *	4.3 [2.1, 14.1]	0.25
IL-13	6.7 [5.5, 10.3]	13.0 [8.0, 17.2] **	0.38
IL-17A	0 [0, 0]	0 [0, 0.42] *	0.34
Monocyte Chemotactic Protein-1 (MCP-1; CCL2)	572 [454, 699] *	522 [394, 692]	0.26
Chemokine Ligand 9 (CXCL9)	8.6 [0, 20.5] **	0 [0, 0]	0.44
Macrophage Inflammatory Protein-1 (MIP-1) Alpha (C-C motif chemokine 3; CCL3)	5.8 [0, 53.8] *	14.6 [0, 45.7]	0.25
Vascular Endothelial Growth Factor A (VEGF-A)	2.6 [1.4, 4.0] **	2.3 [1.4, 3.2]	0.37

Note: SMD—standardized mean difference. Pre-surgery, 69 of 111 participants with patient-reported outcomes also provided blood samples; at the 6-week follow-up, 64 of 75 participants with patient-reported outcomes provided blood samples. Overall, 62 participants provided patient reported outcome data and blood samples and were included in the median difference tests. Both long names and short names for biomarkers are provided for clarity. IQR—interquartile range; PROMIS—Patient Reported Outcome Measurement Information System. * Median was higher than the other time point per paired two-sample signed-rank test without adjustment (*p* < 0.05). ** Median was higher than the other time point per adjusted *p*-values using the Benjamini and Hochberg method (adjusted *p* < 0.05).

**Table 2:** Non-parametric correlation coefficients between patient-reported outcome measures and biomarkers.

Patient-Reported Outcome or Biomarker	Pre-Surgery		Post-Surgery	
	Sleep ^a^	Pain ^b^	Sleep ^a^	Pain ^b^
PROMIS Physical Function	−0.22 **	−0.38 **	−0.06	−0.24
Hepatocyte Growth Factor (HGF)	0.20	0.22	0.11	0.29 *
Interleukin (IL)-1-Beta	0.04	0.38 **	0.15	0.10
IL-2 Receptor	−0.12	−0.01	−0.24	−0.42 ***
IL-4	−0.09	0.30 **	−0.12	0.03
IL-13	−0.07	0.24 *	−0.07	0.08
IL-15	−0.03	0.14	−0.02	−0.05
IL-17A	−0.07	0.26 *	0.10	−0.12
Recombinant Human IP-10 (C-X-C motif chemokine 10; CXCL10)	0.08	0.34 **	0.11	0.16
Monocyte Chemotactic Protein-1 (MCP-1; CCL2)	0.01	0.23	0.14	−0.04
Chemokine Ligand 9 (CXCL9)	0.11	0.29 **	0.01	−0.12
Macrophage Inflammatory Protein-1 (MIP-1) Alpha (C-C motif chemokine 3; CCL3)	0.03	0.28 **	−0.13	0.09
MIP-1 Beta (C-C motif chemokine 4; CCL4)	−0.02	0.31 **	0.05	0.14

Note: ^a^ Pain refers to the pain intensity correlation, ^b^ Sleep refers to the Patient Reported Measurement Information System sleep disturbance correlation. * unadjusted *p* < 0.05, ** false discovery rate ***adjusted *p*-value < 0.05.

## Data Availability

The data that support the findings of this study is available on request from the corresponding author. The raw molecular data for the cytokine chemokine will be available upon request from the corresponding author. All data is freely accessible.

## Supplementary Materials

The following supporting information can be downloaded at: https://www.mdpi.com/article/10.3390/ijms26135959/s1.

## Abbreviations

The following abbreviations are used in this manuscript:

ASA	American Society of Anesthesiologists
PROMIS	Patient-Reported Outcome Measurement Information System
CAT	Computer adaptive testing
IRB	Institutional review board
